# Altering the Natural History of Oligometastatic Prostate Cancer With Local Therapies: Reality Versus Illusion

**DOI:** 10.1200/JOP.2016.018846

**Published:** 2017-01-03

**Authors:** Phuoc T. Tran, Emmanuel S. Antonarakis

**Affiliations:** Johns Hopkins University School of Medicine, Baltimore, MD

In the first definitive treatise on low-volume metastatic cancer by Philip Rubin and Jerold Green, published in 1968 and entitled *Solitary Metastases*, the authors remark that “all too frequently, a solitary metastasis is an illusion rather than a reality.”^1(p232)^ These authors faced similar fundamental questions regarding care for these patients with low-volume metastatic disease: “the clinician is faced with a choice between conservative or radical therapy. What type of surgery, radiotherapy or chemotherapy should be employed?”^1(p3-4)^ So what has changed in the past 50 years? In short, a lot!

With subsequent medical advancements in staging—namely, imaging, and new metastasis-directed therapies such as stereotactic ablative radiotherapy—this formerly illusory oligometastatic state has again become an area of intense interest by cancer physicians.^2^ As originally hypothesized by Hellman and Weichselbaum,^3^ the oligometastatic state is juxtaposed at an intermediate position along the spectrum of cancer progression where local therapies may not only alter their natural history, but also cure men with this metastatic disease state (Fig 1). Preclinical data exist suggesting a unique biology of oligometastases in non–small-cell lung cancer (NSCLC) that has been mechanistically explained by microRNA-mediated attenuation of prometastatic epithelial plasticity programs such as the epithelial-mesenchymal transition.^4^ Similarly, in NSCLC, we now have randomized clinical trial confirmation that local therapies can prolong the progression-free survival of patients with three or fewer metastases after first-line systemic therapy for NSCLC.^5^

**FIG 1.**
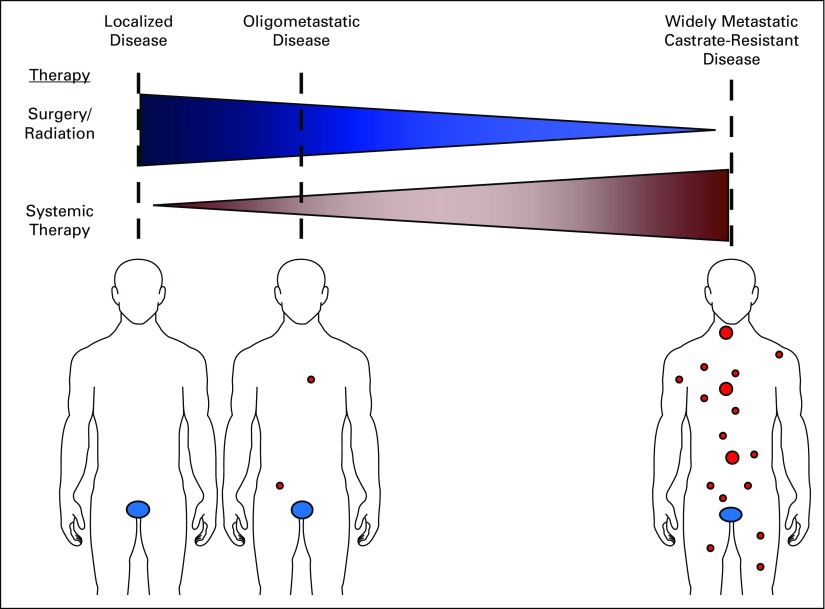
The oligometastatic disease state. The spectrum of malignant disease is represented by blue ovals for primary prostate cancer and red circles for macroscopic metastases. Patients are considered in relation to the putative benefits of local versus systemic therapies. Men in the oligometastatic disease state may benefit from both systemic therapy and local therapies.

It is in this context of renewed excitement and knowledge regarding the oligometastatic state that the accompanying article by Clement and Sweeney^6^ reviews the current clinical standard of care and ongoing clinical trials for men with hormone-sensitive oligometastatic prostate cancer. We commend their efforts to summarize the existing retrospective data and catalog many of the current clinical trials exploring the oligometastatic state in prostate cancer. To expound upon their work, we would like to highlight what we believe are key future outstanding clinical and biologic questions that will be important in the field of hormone-sensitive oligometastatic prostate cancer.

## Defining Oligometastatic Prostate Cancer

First, how does one define oligometastatic prostate cancer? Many unanswered questions still surround the understanding of oligometastasis because the clinical literature offers a wide variety of definitions. However, until a biologic (and likely genomic) understanding exists to define oligometastatic disease, a clinical diagnosis based on up to five radiographically visible metastatic lesions is a reasonable definition.^7^ Until we have such a biologic definition, our working clinical definitions will further be complicated by the stage or risk migration that is occurring with newer and highly sensitive imaging modalities. As commented on by Clement and Sweeney,^6^ we agree that current clinical definitions need to be consistent and based in the context of conventional imaging for which we have the bulk of our clinical data, but at the same time, they should proceed in parallel with the necessary research to develop new advanced imaging techniques. Thus, we will need careful clinical validation of these newer imaging modalities to eventually incorporate them into better definitions of actionable oligometastatic disease.

## Understanding Prevalence

Second, how prevalent is hormone-sensitive oligometastatic prostate cancer? In the modern era with conventional imaging, metastatic prostate cancer as a whole is present in only a minority of newly diagnosed patients, and thus, synchronous or de novo oligometastatic prostate cancer comprises an even smaller subset of these men, likely numbered in the several thousands in the United States. Whether this number will increase because of decreased prostate-specific antigen screening and newer clinical definitions based on advanced imaging modalities is an area of interest. In contrast, metachronous or oligorecurrent (oligoprogressive) prostate cancer comprises a large number of men, possibly the majority of men after failed primary therapy; these men ultimately experience progression to metastatic disease based on the limited number of series that have examined this population.^8-11^ Assuming that these patients are possibly in a curable state before castration resistance develops, we need additional studies to examine this potentially large prevalence in a more robust fashion.

## Biology of Hormone-Sensitive Oligometastatic Prostate Cancer

Third, is there a biologic difference between hormone-sensitive oligometastatic prostate cancer and polymetastatic prostate cancer? The genetic and transcriptomic profiling of hormone-sensitive localized^12^ and metastatic castration-resistant prostate cancer^13^ has been well described recently, but a complimentary data set for the hormone-sensitive oligometastatic state is still unavailable. In addition, exploration of the biology of the oligometastatic state may also uncover biomarkers such as blood-based microRNA signatures or others to further direct care. Having this detailed biologic and genetic classification, assuming that the oligometastatic state is molecularly distinct, would not only afford us a more precise definition to be used clinically, but also provide us an understating of the early metastatic process before the inevitable changes have developed in men with metastatic castration-resistant prostate cancer after the heavy selective forces of multiple systemic treatment regimens. Older models of metastasis portray the unidirectional flow of circulating tumor cells (CTCs) leaving the primary tumor site and seeding a metastasis at a distant site.^14^ However, recent preclinical data using diverse experimental models of breast and colon cancer as well as melanoma suggest that metastasis is a multidirectional process whereby CTCs seed both distant sites as well as the original primary tumor—a process termed self-seeding.^15,16^ Interestingly, genomic lineage tracing of metastases and, in some cases, the primary tumor from a rapid autopsy series of men who died of metastatic castration-resistant prostate cancer suggests that macroscopic metastases represent communal sanctuaries that are composed of prostate cancer cells from many other metastatic sites throughout the body.^17^ These communal sanctuaries are presumably favorable niches that allow prostate cancer cells the ability to gain competence for the development of future macroscopic metastases. These human data from metastatic castration-resistant prostate cancer are consistent with the preclinical concept of self-seeding or a multidirectional flow of CTCs. However, do these provocative data hold true for men with hormone-sensitive oligometastatic cancer? If yes, then metastasis-directed therapies to all macroscopic metastases in patients with oligometastatic disease may eliminate these sanctuaries and alter the natural history of metastatic prostate cancer. Even more provocative, can we convert a polymetastatic incurable biology into an oligometastatic presumably curable biology in a man with prostate cancer? Fortunately, we may have some answers to these important questions in the near future. In addition to the many prospective trials in oligometastatic prostate cancer detailed in the review by Clement and Sweeney,^6^ there is also a Movember Global Action Plan 6^18^ initiative on oligometastatic prostate cancer that is planning to directly investigate many of the biologic and clinical issues identified earlier.

In closing, although much has changed since the time that Rubin and Green published *Solitary Metastases*, some central tenets remain, including that “[t]he management of such patients requires sound judgement and perspective.” ^1(p3)^ We are in full agreement with Clement and Sweeney^6^ that the standard of care for these men is still systemic therapy in the form of androgen deprivation and that any additional local therapies should ideally be implemented in the setting of a clinical trial. However, we are also optimistic that with the data from such prospective trials and efforts like the Movember Global Action Plan 6 initiative and others, we will have more knowledge to benefit these men with oligometastatic prostate cancer in the near future.
